# Evaluation of Mouthwash Containing *Citrus hystrix* DC., *Moringa oleifera* Lam. and *Azadirachta indica* A. Juss. Leaf Extracts on Dental Plaque and Gingivitis

**DOI:** 10.3390/plants10061153

**Published:** 2021-06-06

**Authors:** Watunyoo Buakaew, Rungnapa Pankla Sranujit, Chanai Noysang, Supaporn Sangouam, Nungruthai Suphrom, Yordhathai Thongsri, Pachuen Potup, Kanchana Usuwanthim

**Affiliations:** 1 Cellular and Molecular Immunology Research Unit, Faculty of Allied Health Sciences, Naresuan University, Phitsanulok 65000, Thailand; watunyoob60@nu.ac.th (W.B.); yordhathai.k@gmail.com (Y.T.); pachuenp@nu.ac.th (P.P.); 2 Thai Traditional Medicine College, Rajamangala University of Technology Thanyaburi, Pathum Thani 12130, Thailand; rungnapa_s@rmutt.ac.th (R.P.S.); chanai_n@rmutt.ac.th (C.N.); 3 Faculty of Dentistry, Naresuan University, Phitsanulok 65000, Thailand; supapornsa@nu.ac.th; 4 Department of Chemistry, Faculty of Science and Center of Excellence for Innovation in Chemistry, Naresuan University, Phitsanulok 65000, Thailand; nungruthais@nu.ac.th

**Keywords:** mouthwash, gingivitis, *Citrus hystrix* DC., *Moringa oleifera* Lam., *Azadirachta indica* A. Juss

## Abstract

Oral hygiene and control of microbial plaque biofilm formation are effective methods for preventing gingivitis. Mouthwashes containing leaf extracts of the medicinal plants *Citrus hystrix* DC. (KL), *Moringa oleifera* Lam. (MO) and *Azadirachta indica* A. Juss. (NE) were assessed for oral healthcare and gingivitis adjunctive treatment. Three types of mouthwash were developed; KL, a combination of KL and MO (KL + MO), and a combination of KL, and NE (KL + NE). The mouthwashes were tested in vivo on 47 subjects with gingivitis who were allocated into five groups as (i) placebo, (ii) KL, (iii) KL + MO, (iv) KL + NE, and (v) 0.12% chlorhexidine gluconate (CHX). Participants were instructed to rinse with herbal mouthwash twice daily for two weeks. Gingival index (GI), plaque index (PI), and oral microbial colonies were measured at baseline and 15 days. Results showed that GI and PI of groups (ii)–(iv) significantly decreased over the placebo group, while accumulative reduction percentages of both *Staphylococcus* spp. and *Candida* spp. were found in groups (iii) and (iv). Findings indicated that the herbal mouthwashes reduced GI and PI, and showed potential as oral healthcare products.

## 1. Introduction

Inflammation is a systemic body response against harmful molecules, especially microbial infection or tissue injury. In the early response phase, innate immune cells such as neutrophils, natural killer (NK) cells and macrophages sense the arrival of invading pathogens using a group of cell surface and cytoplasm receptors called pattern recognition receptors (PRRs) [1]. PRRs can detect harmful molecules as pathogen-associated molecular patterns (PAMPs) or host-derived damage-associated molecular patterns (DAMPs), leading to the triggering of intracellular signaling transduction. Various signaling pathways are activated during inflammatory activation, including nuclear factor-κB (NF-κB), resulting in the increase of pro-inflammatory mediator production such as tumor necrosis factor-α (TNF-α), interleukin-1β (IL-1β) and interleukin-6 (IL-6) [2]. These transient inflammatory processes induce the clearance of dangerous molecules and maintain tissue homeostasis in the resolution phase; however, uncontrolled inflammation can become chronic as one of the risk factors contributing to many ailments such as cardiovascular disease, cancer and osteoporosis [3]. DAMPs derived from microorganisms are the main inflammatory activators. Inflammation results from the interaction between immune cells and microbes in different parts of the body. Therefore, controlling inflammation is a key concept in preventing inflammatory-associated disease development.

In the oral cavity, the common inflammatory disease that affects tissue and supportive structures is gingivitis [4]. Gingivitis is a localized reversible plaque-induced inflammation of the gingiva. Depending on the severity of inflammation, gingivitis can trigger a wide range of symptoms, from a slight change in color and edema to spontaneous bleeding of the gingiva [5]. Periodontal disease, as the condition of chronic gingival, bone and ligament inflammation, can develop from uncontrolled gingivitis resulting in irreversible inflammation and tooth loss [6]. Timely control of gingivitis inhibits the progression of periodontal disease. Various risk factors contribute to the occurrence of gingivitis including hormonal fluctuation, drug use, systemic disease and dental plaque [7]. Dental plaque accumulation-induced gingivitis can be observed in individuals with insufficient oral hygiene. Disruption of dental plaque as bacterial biofilm by tooth brushing or flossing is an effective method to maintain normal amounts of oral microbiota and prevent inflammation. However, inadequate timing and inappropriate self-performed practices affect the state of oral hygiene and inflammation. Thus, using the appropriate antimicrobial agents, combined with regular oral care (tooth brushing, flossing), benefits individuals through self-performed hygienic practices. Among the antimicrobial products used in dental care, chlorhexidine gluconate (CHX) is a popular cationic bisbiguanide and broad-spectrum antimicrobial agent showing anti-plaque activity [8]. Mouthwash containing CHX has been widely used in clinical dental care; however, various reports detail the adverse effects of long-term use including teeth surface staining, burning sensation and loss of taste [8]. Thus, finding alternative compounds with reduced side effects would be beneficial to overcome these disadvantages.

Medicinal plants have been identified and reported for their bioactivity in oral care studies [9]. Three medicinal plants with well-known properties against inflammation and microbial infection are *Citrus hystrix* DC. (KL), *Moringa oleifera* Lam. (MO) and *Azadirachta indica* A. Juss. (NE). These three plants are found in several areas of Asia, especially Thailand, and are commonly used in traditional medicine and routine food. Our previous studies found that crude extracts containing bioactive compounds of KL and MO leaves showed anti-inflammatory activities by inhibiting inflammasome and regulating the releasing of pro-inflammatory mediators of lipopolysaccharide (LPS)-induced human macrophages that are regulated through the NF-κB pathway [10,11]. We found that KL leaf extract prevented biofilm formation by inhibiting the expression of biofilm-associated genes of the oral bacterium *Streptococcus mutans* [12]. Moreover, NE leaf extract showed protection against cigarette smoke and LPS-induced pulmonary inflammation in mice [13].

Although numerous potential biological activities, including anti-inflammation and antimicrobial ability, have been reported from KL leaf extract, information is still lacking regarding treatment efficiency in the human model, especially for mouthwash development to combat gingivitis. Moreover, finding a choice to ameliorate CHX side effects would be beneficial for maintaining oral hygiene. Thus, here, we developed and evaluated the efficacy of mouthwash containing ethanolic leaf extracts of KL, MO, and NE on the gingival index (GI), plaque index (PI), and oral microbe colony count in gingivitis subjects.

## 2. Results

### 2.1. Total Phenolic Content

**Figure 1:**
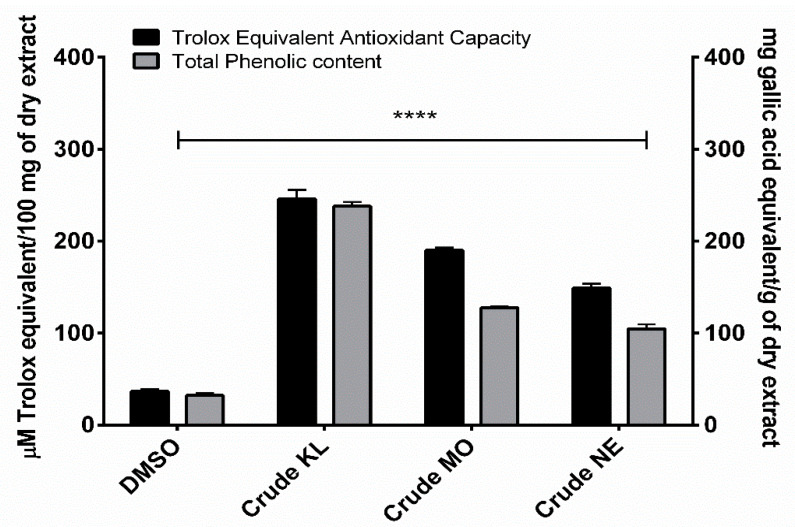
Trolox equivalent antioxidant capacity and total phenolic content of *Citrus hystrix* DC. (KL), *Moringa oleifera* Lam. (MO) and *Azadirachta indica* A. Juss. (NE) crude leaf ethanolic extracts. Results are expressed as Trolox equivalent (µM) and gallic acid equivalent (mg) for antioxidant and total phenolic contents, respectively. Data are presented as mean ± SD. * *p* < 0.05; ** *p* < 0.01; *** *p* < 0.001; **** *p* < 0.0001 compared to DMSO: dimethyl sulfoxide.

Phenolic contents of ethanolic leaf extracts were measured using Folin-Ciocalteu reagent, with results expressed as mg of gallic acid equivalent (GAE) per gram of dry weight extract. Amount of total phenolic content in the leaves of the three plants ranged from 104.62 to 237.97 GAE/g. KL showed the highest phenolic content (237.97 ± 8.10 mg/GAE) followed by MO (127.710 ± 2.29 mg/GAE) and NE (104.62 ± 8.47 mg/GAE), as shown in Figure 1.

### 2.2. Trolox Equivalent Antioxidant Capacity (TEAC)

Ethanolic extracts of the three plants showed similar TEAC profiles to total phenolic contents. KL had the greatest TEAC (245.99 ± 17.01 µM) with MO (189.93 ± 5.24 µM) and NE (149.04 ± 7.99 µM) of Trolox equivalent, as shown in Figure 1.

### 2.3. GC-MS Analysis of Chemical Constituents in Crude Leaf Ethanolic Extracts

**Figure 2:**
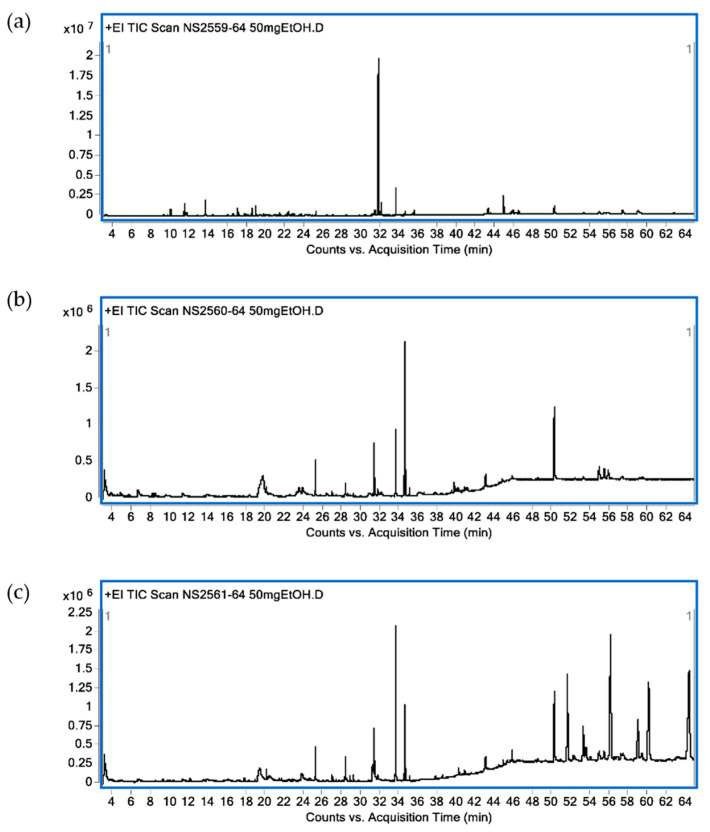
Chromatogram from GC-MS analysis. *C. hystrix* DC. (**a**), *Moringa oleifera* Lam. (**b**) and *Azadirachta indica* A. Juss. (**c**).

Identified bioactive compounds present in leaf ethanolic extracts obtained from KL, MO and NE as terpenoid constituents are shown in Table 1. In KL leaf extract, the main identified compounds were terpenoids (86.89%) composed of monoterpenes (13.50%), sesquiterpenes (6.39%), diterpenes (57.34%) and triterpenes (11.59%). The three major terpenoids were tetraprenol (47.42%), phytol (6.57%) and citronellol (4.28%). Terpenoids (16.42%) were found in MO leaf extract as diterpenes (9.81%) and triterpenes (6.61%). The three main terpenoid constituents were phytol (8.39%), β-sitosterol (3.79%) and β-amyrin (2.82%). For NE ethanolic leaf extract, terpenoids accounted for 23.32% with sesquiterpenes (0.15%), diterpenes (9.13%), triterpenes (5.48%) and tetraterpene (8.56%). The major active terpenes were astaxanthin (8.56%), phytol (7.56%) and stigmasterol (3.97%). A GC-MS chromatogram of all plant extracts is shown in Figure 2.

### 2.4. Quality Control Assessment of Formulated Mouthwashes

Ethanolic leaf extracts of KL, MO and NE were used to formulate three herbal mouthwashes containing KL ethanolic extract alone, a combination of KL and MO, and a combination of KL and NE. The three formulated mouthwashes were assessed for quality control following the food and drug administration (FDA) of Thailand mouthwash formulation standard for appearance, pH, heavy metal content and microorganisms. Results were in the acceptable specification range, as shown in Table 2.

### 2.5. Baseline Information of Participants

**Figure 3:**
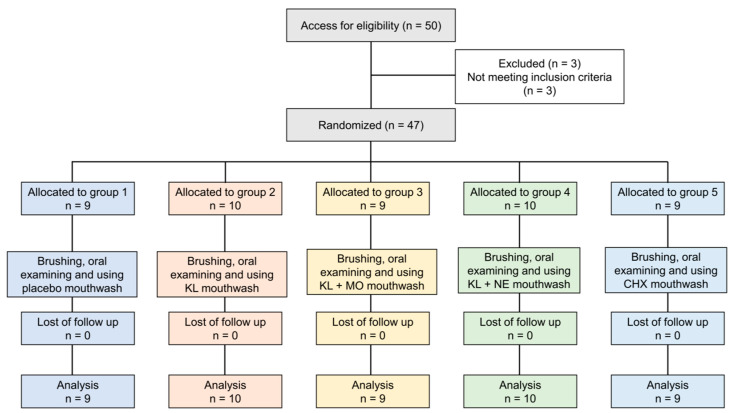
Flow chart of the study.

Fifty participants were recruited. Three people did not meet the inclusion criteria and were excluded, as shown in Figure 3. The remaining 47 eligible volunteers were assessed for baseline information including age, sex and mean GI and PI scores, as shown in Table 3. In total, 47 subjects (31 females, 16 males) had a mean age of 23.51 (±6.60) with ages ranging from 20 to 48. Overall mean scores of PI and GI at baseline were 1.12 (±0.55) and 1.42 (±0.65), respectively. There were no statistically significant differences between age and baseline scores of PI and GI among the five treatment groups.

### 2.6. GI and PI Scores from Baseline to Day 15

**Figure 4:**
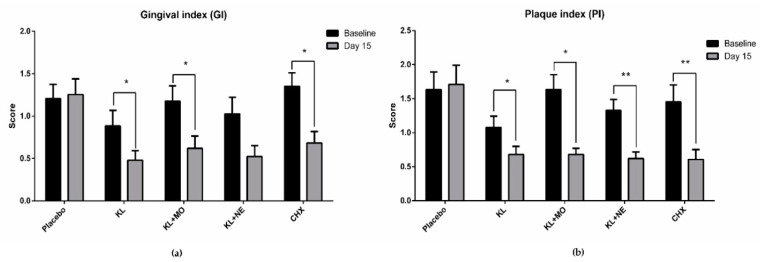
Intragroup comparison of gingival index (**a**) and plaque index (**b**) scores between baseline and day 15 after mouthwash usage by the Wilcoxon signed-rank test. Data are presented as mean ± SD. * *p* < 0.05; ** *p* < 0.01; *** *p* < 0.001; **** *p* < 0.0001.

GI and PI scores from baseline to day 15 in each experimental group are shown in Figure 4. Intragroup comparison of GI scores on day 15 in the KL, KL + MO and CHX groups recorded significant decrease from the baseline, as shown in Figure 4a. Reduction of PI score showed a similar trend to GI score that included KL+NE, as shown in Figure 4b.

### 2.7. Change in Number of Oral Microbial Colonies

Oral rinses from all subjects were cultured on agar plates and numbers of microbial colonies were counted. The effect of mouthwash was evaluated on *Staphylococcus* spp. and *Candida* spp. growth. Average colony count and accumulative reduction percentage between periods are shown in Table 4. For colonies of *Staphylococcus* spp., the CHX group showed the highest reduction percentage at 54.11 followed by KL + MO, KL + NE, KL and placebo groups, respectively. Highest reduction percentage of *Candida* spp. colony was found in the KL + NE group followed by KL + MO and CHX groups, respectively.

## 3. Discussion

Dental plaque accumulation is one of the key factors contributing to gingivitis development. Plaque formation at the gingival margin induces inflammation called plaque-induced gingivitis and prevalence can be found in all ages of dentate populations [7]. Common signs of plaque-induced gingivitis include erythema, bleeding and tenderness depending on individual severity. Untreated gingivitis increases the risk of periodontitis progression and eventually leads to tooth loss. Chlorhexidine (CHX) mouthwash is an effective choice for controlling dental plaque formation; however, long-term side effects may limit use in oral care routines.

Accordingly, seeking a safe adjunctive aid for daily self-oral hygiene would benefit individuals and lower the risk of serious periodontal progression. Medicinal plants have long been used to improve human health and oral healthcare. Many plants have shown effective action to remedy gingivitis [9]. This study focused on three medicinal plants as KL, MO and NE for mouthwash formulation. Results of GI and PI scores in participants after 14-day usage of mouthwashes containing plant extracts showed a significant decrease compared to the baseline (Figure 4). Although GI score in the KL + NE group was not statistically different, the trend of reduction was similar to both KL and KL+MO groups.

Results of GC-MS analysis indicated that volatile substances in the terpenoids group may be responsible for the anti-inflammatory activity of these medicinal plants. Several reports document the anti-inflammatory and antimicrobial effects of volatile terpenes and terpenoids derived from plants [14,15]. Phytol, an acyclic diterpene alcohol and a constituent of chlorophyll was identified in the ethanolic leaf extracts of all three plants. This diterpene has potential bioactivities including antioxidant activity [16], anti-inflammation [17] and antimicrobial effects [18]. The anti-inflammatory activity mechanism of phytol may be explained by targeting and downregulating the molecules in mitogen-activated protein kinases (MAPK) and nuclear factor-kB (NF-κB) signaling pathways [19]. Other major active terpenoid compounds identified from KL leaf extract included citronellal, citronellol and lupeol. By contrast, fewer volatile compounds were identified in MO and NE extracts compared to KL. This suggested KL as a potential source of terpenoids.

To evaluate the antimicrobial activity of the formulated mouthwashes, the spread plate method was performed to observe growth of oral microorganisms. Some reports suggested that colonization of *Staphylococcus* spp. may be associated with the prevalence of gingivitis [20,21]. Here, we found *Staphylococcus* spp. in the oral rinse samples. All plant extract mouthwashes increased the accumulative reduction percentage of microbial colonies from the baseline, as shown in Table 4. Increasing accumulative reduction percentage of *Staphylococcus* spp. was observed in all groups, including the placebo group. One possible reason for this reduction might be the Hawthorne effect [22] that contributes to behavior change of participants due to awareness of being part of clinical trials, while reduction of *Staphylococcus* spp. gave no change in PI score in the placebo group (Figure 4). One explanation of this scenario could be that dental plaque biofilm is mainly composed of cariogenic bacteria such as *S. mutans, Lactobacillus rhamnosus* and *Fusobacterium nucleatum* [23]. Thus, the active compounds in KL, MO and NE may act synergistically when combined with tooth brushing to reduce dental biofilm formation by cariogenic bacteria. Performing tooth brushing alone might not be sufficient to decrease *Staphylococcus* spp. colonization in the oral cavity. Other factors such as performing oral hygiene practice and diets may contribute to different numbers of *Staphylococcus* spp. and other oral microorganism colonization in individuals.

*Candida* spp., is known as a fungus that can cause various types of oral pathology including oral candidiasis in immunocompromised hosts [24]. Due to the inclusion/exclusion criteria, all subjects in this study were immunocompetent, which resulted in few *Candida* spp. in their oral rinse samples. One explanation of the antimicrobial mechanism is based on the ability of phytol to induce oxidative stress by increasing intracellular reactive oxygen species (ROS) accumulation as well as transient depletion of nicotinamide adenine dinucleotide (NADH), leading to DNA damage and eventually cell death [18].

Apart from the identified volatile components in this study, several reports identified bioactive compounds and bioactivity of each plant depending on the extraction method and technique of identification. The major chemical constituents of NE leaves are nimbin, nimbanene and 6-desacetylnimbinene [25]. Moreover, quercetin and β-sitosterol from the leaves showed antibacterial and antifungal activities [26]. In addition to the in vitro study, the NE extract improved GI and PI in clinical trials [27]. For KL, aqueous extract from the leaves showed activity in preventing biofilm formation in vitro, as well as inhibiting the expression of biofilm-associated genes of the oral bacterium *Streptococcus mutans* [12]. Another report on the chemical components of KL essential oil from leaves using gas chromatography/mass spectrometry (GC/MS) detected monoterpenoids such as β-citronellal, terpinen-4-ol, β-citronellol and 1,8-cineole, while MO extract inhibited the growth of candidate oral microbes [28]. Apart from antimicrobial activity, ethyl acetate extract from MO leaves exhibited anti-inflammatory activity in cigarette smoke-stimulated human macrophages by targeting the NF-κB signaling pathway [29]. Active compounds in the leaf extract were identified as 3,4-methyleneazelaic acid, (2S)-2-phenylmethoxybutane-1,4-diol and 3-hydroxy-β-ionone [11].

Limitations of this study are lack of quantitative analysis in formulated mouthwashes and crude extracts related to content of active compounds, and no data of genetic heterogeneity of each plant utilized in mouthwashes. Since the genetic variability affects the biochemical profiling in plants including *C. hystrix* [30], *A. indica* [31], and *M. oleifera* [32]. However, chemical compounds analysis using GC-MS were performed in the study may not represent the entire chemical and active compound profile of plant and may be different from related species due to geography and environment. Another limitation is due to the inclusion of 47 participants from 50. Three volunteers using antibiotics according to exclusion criteria were eliminated from the study.

## 4. Materials and Methods

### 4.1. Plant Preparation and Extraction

Powdered leaves of KL, (COA Lot. No. 250818), MO (COA Lot. No. 5534) and NE (COA Lot. No. 021018) were obtained from Khaolaor Company (Samut Prakan, Thailand). Briefly, 1700 g of KL powder was macerated in 5100 mL of 95% ethanol for three days, with 1000 g of MO powder in 5100 mL and 1000 g of NE powder in 3000 mL 95% ethanol for seven days. All extracts were filtered using 0.45-micron filter paper and evaporated by a rotary evaporator at 35 °C. Three crude extracts were obtained and percentage yields (% *w*/*w* dried powder) were calculated as 133 g (6.65% yield) of KL, 106.75 g (10.68% yield) of MO and 89 g (8.9% yield) of NE ethanolic extracts. All the leaf extracts were determined for ethanol residual solvent using gas chromatography-mass spectrometry (GC-MS) with headspace technique. Mean residual solvent percentage in the extracts ranged 5.14 ± 0.85% (*w*/*v*).

### 4.2. Determination of Total Phenolic Content by Folin-Ciocalcteu Method

Total phenolic contents of ethanolic extracts from KL, MO and NE were evaluated using the Folin-Ciocalcteu method as previously described, with slight modifications [33]. Briefly, 2.5 µL of all extracts (100 mg/mL) and serially diluted gallic acid were added to a 96-well plate. Five microliters of Folin-Ciocalteu reagent (10% *v*/*v*) were added to each well, followed by 90 µL of Na_2_CO_3_ (2% *w*/*v*). The plate was incubated at 50 °C for 1 h. The absorbance of the mixture was measured at 750 nm.

### 4.3. Determination of Trolox Equivalent Antioxidant Capacity (TEAC)

The protocol for Trolox-equivalent antioxidant capacity (TEAC) was determined based on 1,1-diphenyl-2-picrylhydrazyl (DPPH) scavenging reaction, similar to the procedure from a previous report with some modifications [34]. Briefly, 5 µL of all extracts and serially diluted 0.2 mM Trolox were added to a 96-well plate. Then, 50 µL of DPPH in methanol (1:5) was added and incubated in the dark at room temperature for 2 h. Sample absorbance was measured at 517 nm.

### 4.4. Gas Chromatography-Mass Spectrometry Analysis (GC-MS)

GC-MS analyses of volatile compounds from the three crude ethanolic leaf extracts of KL, MO and NE were performed using a Hewlett Packard Gas Chromatograph model 6890 (Agilent Technologies, Palo Alto, CA, USA) equipped with a mass selective detector. The chemical components in all extracts were separated in a silica capillary Hewlett Packard HP-5 (5% phenyl methyl siloxane) column (30 m × 0.25 mm i.d., 0.25 µm film thickness). Pure helium was used as the carrier gas with a constant flow rate at 13.7 mL/min. Initial temperature was set at 250 °C with split ratio mode at ratio 10:1 and 1 µL injector volume. The oven temperature was started at 70 °C for 3 min with increasing rate of 5 °C/min to 280 °C and holding time of about 20 min. Transfer temperature was set at 280 °C and mass detection ranges were set from 50 to 700 amu in full scan.

Identification of volatile compounds was performed by computer matching of their recorded mass spectra fragmentation patterns in the Wiley 7n MS spectral library or with previously published data. Identified compounds from the three plants were expressed as percentages based on the peak areas produced in the chromatogram.

### 4.5. Study Design

This study was designed as a single-blind, randomized, placebo-controlled clinical trial. Study protocol was approved by the Human Ethics Committee (IRB no. 1065/61) at Naresuan University. The enrolled volunteers provided written informed consent following the protocol of the Declaration of Helsinki. Each participant was identified using a code and randomly allocated to one of five experimental groups based on simple random sampling using computer-based randomization software. All participants were identified using a three-number code. The first digit indicated the allocated treatment group from 1–5, while the last two digits indicated the number of the participant. The code explanation was blinded to the examiner and all participants. Identifying data in this study could be accessed by the main researcher only. Subjects in each group were assigned to use different types of mouthwash. The study protocol included assessment of GI and PI scores, as well as the number of oral microbial colonies at the first visit and after mouthwash usage for 14 days.

Sample size was calculated by the following formula where *n* is required sample size; *Z* is a constant: *Z_α_* (1.96) and *Z*_1−*β*_ (0.84); σ is a standard deviation (0.8) and Δ is the difference in effects of two interventions (1) [35]. From this calculation, the number of participants required in this study is 50.

(1) $$n=\frac{2{\left(Z_{\alpha }+Z_{1-\beta }\right)}^{2}\sigma^{2}}{\Delta^{2}}$$

### 4.6. Participants

Fifty healthy volunteers aged between 20 and 48 (median 21 ± 6.6) from Naresuan University were recruited for the study. All the subjects were screened for suitability by the research team. A total of 47 participants met the inclusion criteria and were included in this study. Inclusion criteria included (i) 18–60 years old, (ii) diagnosed with gingivitis with gingival index (GI) and plaque index (PI), (iii) good general health, and (iv) able to communicate in Thai fluently.

Exclusion criteria included (i) wearing orthodontic appliances or implants, (ii) presence of periodontitis, (iii) use of drugs including antibiotics, anti-inflammatory, anticoagulants, anticonvulsants, immunosuppressants and chemotherapy drugs within the past six months, (iv) pathologies of diabetes, xerostomia, oral cancer or receiving radiation therapy, (v) smoker, (vi) pregnant or breastfeeding, (vii) allergic to herbs or chemical constituents in mouthwash, (viii) severe alcoholism, and (ix) presence of mucous membrane pemphigoid. Eligible participants received oral and written information concerning the treatment products and research objectives. All subjects could withdraw from the study at any time. The study flow chart is shown in Figure 3.

### 4.7. Interventions

On the first day, all qualified subjects were assigned to brush their teeth 5 h before gargling with normal saline for 1 min. The oral rinse samples were collected and kept at 4 °C. The baselines of GI and PI scores of individual subjects were evaluated by a dentist using a periodontal probe and 6% erythrosine solution staining, respectively. After the assessment, all subjects were randomly assigned to one of the following five groups: group 1: placebo mouthwash, group 2: Kaffir lime (KL) leaf extract mouthwash, group 3: KL with Moringa (MO) leaf extract mouthwash, group 4: KL with Neem (NE) leaf extract mouthwash and group 5: 0.12% Chlorhexidine (CHX) mouthwash. The participants were asked to gargle with the mouthwash for 14 days in the amount of 10–15 mL twice daily for 30 s after brushing their teeth. Subsequent rinsing with water, drinking and eating were not allowed within 15 min. Use of commercial mouthwash during the study was prohibited.

To examine the change of gingival condition in terms of inflammation and plaque buildup, the gingival index (GI) and plaque index (PI) scoring system of Löe-Silness was used [5]. Gingival inflammation was examined based on the GI system, where inflammation of the gum is graded into four scales from 0 (normal) to 3 (severe gingival inflammation). To evaluate the formation of plaque, a 6% erythrosine solution was used, with scores from 0 (no debris or stain present) to 3 (soft debris covering more than two-thirds of the exposed tooth surface).

### 4.8. Mouthwash Descriptions

In this study, five types of mouthwash were used: (i) placebo mouthwash, (ii) KL mouthwash, (iii) KL and MO mouthwash, (iv) KL and NE mouthwash, and (v) 0.12% Chlorhexidine mouthwash. KL mouthwash contained an ethanolic extract of KL leaves (0.03% *w*/*w*). KL and MO mouthwash contained ethanolic extracts of KL leaves (0.015% *w*/*w*) and MO leaves (0.015% *w*/*w*). KL and NE mouthwash contained ethanolic extracts of KL leaves (0.015% *w*/*w*) and NE leaves (0.015% *w*/*w*). Common ingredients of mouthwash in groups (i)–(iv) included water, propylene glycol, glycerin, sorbitol, sucralose, flavoring agents, sodium benzoate and coloring agents. Group (v) was commercial 0.12% Chlorhexidine gluconate mouthwash (C-20 Chlorhexidine antiseptic mouthwash, Edwards, Bangkok, Thailand).

### 4.9. Assessment and Outcome

The assessment protocol was divided into two parts as the baseline and day 15 assessments. Baseline assessment was conducted during the first visit, followed by day 15 assessment after two weeks. At each visit, the subjects were instructed to brush their teeth and not to eat or drink anything, except for water, 5 h before the assessment. The assessment included gingival index (GI) and plaque index (PI) measurement by a trained and calibrated dentist.

The GI score assessed the severity of gingivitis by examining qualitative change of gingival tissue using a mouth mirror. A periodontal probe was applied to four areas of the gumline of six selected index teeth. After 15 s, bleeding of each area was recorded according to the following scale: 0, absence of bleeding, 1, slight change in color, slight edema, no bleeding on probing, 2, redness, edema and glazing, bleeding on probing and 3, marked redness and edema, ulceration, tendency to spontaneous bleeding.

The PI score was assessed by staining with a 6% erythrosine solution. After 15 s, the subjects were instructed to rinse their mouths with water, and a mouth mirror was used to observe plaque staining on four areas of six selected index teeth. The score was graded according to the following scale: 0, no debris or staining present, 1, soft debris not covering more than 1/3 of the tooth surface, 2, soft debris covering more than 1/3 but not more than 2/3 of the tooth surface and 3, soft debris covering more than 2/3 of the tooth surface.

Microbial colony count were evaluated using the spread plate method. Oral rinse samples collected from the subjects were vortexed for 10 s. Then, 0.1 mL of each sample was placed on two selective mediums as (i) Mannitol Salt Agar (MSA) and (ii) HiCrome™ Candida Differential Agar. Sample agar plates were incubated at 35 ± 2 °C for 20–24 h. Colonies of *Staphylococcus* and *Candida* species were identified based on morphology and then confirmed by matrix-assisted laser desorption/ionization-time of flight mass spectrometry (MALDI-TOF MS). Image J software with a colony counter plugin was used to count the colonies. The primary efficacy outcome was different for mean GI and PI. The secondary outcome was the reduction of microbial colony count from baseline to day 15.

### 4.10. Statistical Analysis

SPSS software version 26 (Released 2019. IBM SPSS Statistics for Windows, Version 26.0., IBM Corp, Armonk, NY, USA) and GraphPad Prism Software version 6 (GraphPad Software Inc., San Diego, CA, USA) were used to analyze the study parameters. A one-way ANOVA followed by Tukey’s multiple comparison post-hoc test was used to calculate the statistical difference of means from the in vitro experiment. The Shapiro-Wilk test was used to evaluate the distribution of the data. The Wilcoxon signed rank test and paired-sample *t*-test were used to compare differences of the data between baseline and day 15. The Kruskal-Wallis test was used to compare mean differences among the five groups. A value of *p* < 0.05 was considered statistically significant.

## 5. Conclusions

In this in vivo study of mouthwashes containing KL, KL + MO, and KL + NE showed significant decreases in both GI and PI scores. Increasing accumulative reduction percentage of both *Staphylococcus* spp. and *Candida* spp. after 15 days of usage was observed in KL + MO and KL + NE groups compared to the baseline. Findings suggested that mouthwashes containing KL, MO and NE leaf extracts had potential for alleviating dental plaque formation and reducing inflammation of the gingiva as an alternative oral care product for adjunctive treatment in microbial-induced gingivitis.

## Figures and Tables

**Table 1:** Chemical constituents identified by GC-MS from *C. hystrix*, *M. oleifera* and *A. indica* ethanolic extracts.

RT (min)	Identified Compounds	Classification	Relative Area (%)		
			*C. hystrix*	*M. oleifera*	*A. indica*
9.29	*trans*-Linalool oxide	Monoterpene	0.26	-	-
10.00	β-Linalool	Monoterpene	1.61	-	-
11.36	Isopulegol	Monoterpene	1.06	-	-
11.51	Citronellal	Monoterpene	3.16	-	-
11.68	Isopregol	Monoterpene	0.71	-	-
13.63	Citronellol	Monoterpene	4.28	-	-
14.37	*cis*-Geraniol	Monoterpene	0.17	-	-
16.01	Citronellic acid	Monoterpene	0.31	-	-
16.56	2-(2-Hydroxy-2-propanyl)-5-methylcyclohexanol	Monoterpene	0.59	-	-
17.02	Citronellol acetate	Monoterpene	1.94	-	-
17.15	Menthoglycol	Monoterpene	0.25	-	-
17.75	Copaene	Sesquiterpene	0.50	-	0.15
17.97	Ethoxycitronellal	Monoterpene	0.18	-	-
18.51	(2*E*)-1-Ethoxy-3,7-dimethyl-2,6-octadiene	Monoterpene	1.88	-	-
18.91	β-Caryophyllene	Sesquiterpene	2.64	-	-
19.33	Sucrose	Disaccharide	0.75	24.20	4.66
19.77	α-Caryophyllene	Sesquiterpene	0.40	-	-
20.1	1-Dodecanol	Fatty alcohol	-	0.91	0.58
21.43	δ-Cadinene	Sesquiterpene	0.78	-	-
22.05	Elemol	Sesquiterpene	0.20	-	-
22.31	*trans*-Nerolidol	Sesquiterpene	1.08	-	-
22.77	(-)-Spathulenol	Sesquiterpene	0.38	-	-
22.92	Caryophyllene oxide	Sesquiterpene	0.41	-	-
23.70	Ethyl α-d-glucopyranoside	Glycoside	-	1.59	1.31
25.20	Dodecyl acrylate	Fatty ester	0.95	4.24	1.70
28.30	Phytol acetate	Diterpene	-	1.42	1.26
29.17	3,7,11,15-Tetramethyl-2-hexadecen-1-ol	Diterpene	-	-	0.31
30.47	Isophytol	Diterpene	0.13	-	-
31.35	Ethyl palmitate	Fatty acid ethyl ester	1.21	6.09	2.45
31.72	Lauryl 3-mercaptopropionate	Fatty acid ester	-	0.59	0.21
31.75	Tetraprenol	Diterpene	47.42	-	-
32.00	*trans*-Geranylgeraniol	Diterpene	3.22	-	-
33.57	Phytol	Diterpene	6.57	8.39	7.56
34.44	Ethyl-9,12-octadecadienoate	Fatty acid ethyl ester	0.32	2.33	0.30
34.56	Ethyl linolenate	Fatty acid ethyl ester	1.05	18.89	3.85
34.99	Ethyl stearate	Fatty acid ethyl ester	0.22	0.88	0.23
40.15	Glycerol β-palmitate	Fatty acid ester	-	-	0.46
42.97	α-Glyceryl linolenate	Fatty acid ester	-	1.82	1.01
43.5	4-(2,3-Dihydroxy-3-methylbutoxy)furo(3,2-g)chromen-7-one	Furanocoumarin	0.52	-	-
44.84	Squalene	Triterpene	4.12	-	0.21
50.14	α-Tocopherol	Triterpene	3.26	14.80	5.87
51.52	Astaxanthin	Tetraterpene	-	-	8.56
53.2	Stigmasterol	Sterol	0.54	-	3.97
54.83	β-Sitosterol	Sterol	1.57	3.79	1.29
55.36	24-*n*-propylidenecholesterol	Sterol	-	3.11	0.66
55.44	Dihydrolanosterol	Triterpene	0.93	-	-
55.82	β-Amyrin	Triterpene	-	2.82	-
56.05	Phorbol	Diterpene	-	-	16.81
57.31	Lupeol	Triterpene	2.10	-	-
58.91	Olean-12-ene-3,15,16,21,22,28-hexol	Triterpene	-	-	5.37
58.92	Cycloeucalenol acetate	Triterpene	2.32	-	-
60.03	14,15β-epoxy-3β,5-dihydroxy-5β-Bufa-20,22-dienolide	Diterpene	-	-	12.28
64.25	Olean-12-ene-3,16,21,22,23,28-hexol	Triterpene	-	-	18.94

**Table 2:** The results of quality control testing of formulated mouthwashes.

Test Parameters	Test Procedure	Specification	Mouthwash		
			KL	KL + MO	KL + NE
Turbidity	Organoleptic	Transparent	Transparent	Transparent	Transparent
Color	Organoleptic	Green	Green	Green	Green
pH	pH Meter	5.5–8.5	6.6	6.7	6.8
Lead (Pb)	Based on AOAC (2016) method	<10.0 ppm	Not detected	Not detected	Not detected
Arsenic (As)	Based on AOAC (2016) method	<4.0 ppm	<0.5 ppm	Not detected	<0.5 ppm
Mercury (Hg)	Based on AOAC (2016) method	<0.5 ppm	Not detected	Not detected	Not detected
*Clostridium* spp.	USP 41 <62>	Absent/1 g.	Absent/1 g.	Absent/1 g.	Absent/1 g.
*Staphylococcus aureus*	USP 41 <62>	Absent/1 g.	Absent/1 g.	Absent/1 g.	Absent/1 g.
*Pseudomonas aeruginosa*	USP 41 <62>	Absent/1 g.	Absent/1 g.	Absent/1 g.	Absent/1 g.
*Candida albicans*	USP 41 <62>	Absent/1 g.	Absent/1 g.	Absent/1 g.	Absent/1 g.

AOAC: The Association of Official Agricultural Chemists, USP: The United States Pharmacopeia.

**Table 3:** The characteristics data of subjects and baseline clinical parameters (mean ± SD).

Parameters	Placebo (*n* = 9)	KL (*n* = 10)	KL + MO (*n* = 9)	KL + NE (*n* = 10)	CHX (*n* = 9)	*p*-Value
Age (years)	23.11 ± 4.43	21.40 ± 1.71	22.89 ± 5.33	23.90 ± 8.14	26.44 ± 10.42	0.6749 ^a^
GI score	1.21 ± 0.50	0.88 ± 0.58	1.18 ± 0.54	1.03 ± 0.63	1.35 ± 0.48	0.228 ^a^
PI score	1.63 ± 0.77	1.08 ± 0.51	1.63 ± 0.65	1.33 ± 0.52	1.45 ± 0.74	0.371 ^a^
Sex, Female/Male	8/1	6/4	6/3	6/4	5/4	

^a^ Kruskal-Wallis test. A *p*-value < 0.05 was considered statistically significant.

**Table 4:** The number of microbial count (×10^8^) in mean ± SD and accumulative reduction percentage comparing to baseline.

Microorganisms	Intervals	Placebo	KL	KL + MO	KL + NE	CHX
*Staphylococcus* spp.	Baseline	21 ± 38	91 ± 188	62 ± 69	132 ± 148	28 ± 39
	Day 15	78 ± 114	109 ± 176	46 ± 87	83 ± 161	27 ± 48
Accumulative reduction percentage		12.66	30.14	45.53	33.23	54.11
*Candida* spp.	Baseline	1 ± 0	0 ± 1	1 ± 2	1 ± 1	8 ± 23
	Day 15	1 ± 0	1 ± 3	0 ± 0	1 ± 1	2 ± 5
Accumulative reduction percentage		0	0	20	35.83	17.63

## Data Availability

The data presented in this study are available within the article.
